# The Role of Small Extracellular Vesicles and MicroRNAs in the Diagnosis and Treatment of Allergic Rhinitis and Nasal Polyps

**DOI:** 10.1155/2022/4428617

**Published:** 2022-06-16

**Authors:** Yiting Liu, Jichao Sha, Cuida Meng, Dongdong Zhu

**Affiliations:** ^1^Department of Otolaryngology Head and Neck Surgery, China-Japan Union Hospital of Jilin University, Changchun, China; ^2^Jilin Provincial Key Laboratory of Precise Diagnosis and Treatment of Upper Airway Allergic Diseases, China

## Abstract

Allergic rhinitis and nasal polyps are common otorhinolaryngological diseases. Small extracellular vesicles and microRNAs have recently become major research topics of interest due to their key regulatory roles in cancer, inflammation, and various diseases. Although very detailed and in-depth studies on the pathogenesis and pathophysiology of allergic rhinitis and nasal polyps have been conducted, few studies have assessed the regulatory effects of exosomes and microRNAs on allergic rhinitis and nasal polyps. This paper reviews the studies on small extracellular vesicles and microRNAs in allergic rhinitis and nasal polyps conducted in recent years and focuses on the regulation of small extracellular vesicles and microRNAs in allergic rhinitis and nasal polyps with the aim of providing insights for the future diagnosis and treatment of allergic rhinitis and nasal polyps.

## 1. Introduction

Allergic rhinitis (AR) is clinically defined in the 2008 Allergic Rhinitis and its Impact on Asthma (ARIA) Guidelines as “a symptomatic disorder of the nose induced after allergen exposure by an IgE-mediated inflammation” [1]. Epidemiologic studies have revealed that the prevalence of AR has increased progressively in developed countries, and AR currently affects up to 40% of the population worldwide. The clinical manifestations include sneezing and runny or stuffy nose. Patients with AR can develop asthma and other diseases [2, 3]. Nasal polyps (NPs) are common otolaryngological findings in adults [4]. NPs can be observed by nasal endoscopy as soft and smooth masses that resemble water droplets or grapes in the upper and middle nasal passages of patients [5, 6]. NPs are mostly treated by surgery, but the recurrence rate in most patients is high. At present, the occurrence of AR and NPs seriously affects the work, education, and life of patients, and the treatment of these two diseases places a heavy economic burden on society [3, 7]. Both diseases are chronic nasal inflammatory diseases caused by the inflammatory response induced by pathogenic chronic stimulation. The two diseases have a similar pathogenesis, such as destruction of the nasal epithelial barrier and physical barrier and the intensification of Th2 cell differentiation [6, 8].

Small extracellular vesicles (sEVs) are lipid bilayer vesicles with a maximum diameter of 150 nm that are secreted by a variety of cells through the processes of endocytosis, fusion, and exocytosis [9]. SEVs are mostly derived from haematopoietic stem cells, epithelial cells, and tumour cells and exist in various human tissues and body fluids [10–12]. Due to their lipid bilayer structure, sEVs can carry microRNAs (miRNAs), DNA, and other substances. SEVs are generated through the physiological processes of endocytosis, fusion, and exocytosis and can be found throughout the body. SEVs can affect signal transmission, mediate information exchange between cells, and play particular biological roles [13–15]. The clinical research on sEVs has primarily focused on metabolic function. However, with the development of medical technology and increased knowledge, researchers have shown that sEVs can function as conduits of intercellular communication and have utility in the diagnosis, treatment, and prognosis evaluation of a series of diseases [9, 16]. Airway inflammation is involved in both NPs and AR. A previous study reported that intensification of the inflammatory reaction increases the level of secreted sEVs, which further promotes progression of the inflammatory reaction [17–19]. This review aims to provide new methods for the diagnosis, treatment, and prognosis of airway inflammatory diseases by summarizing the role of sEVs in AR and NPs.

miRNAs are endogenous RNAs that regulate gene expression in a posttranscriptional manner; these RNAs are widely expressed in eukaryotic cells and play important roles in biological activities [20]. miRNAs not only affect cell proliferation, differentiation, and apoptosis but also regulate tissue development, the immune response, and tumour formation [21]. The generation of primary miRNA (pri-miRNA) is catalysed by ribonuclease II and prompts the intron sequence to adopt a hairpin ring-like structure [22]. Subsequently, the hairpin ring is removed from the pri-miRNA by the RNase III enzyme Drosha in the nucleus to generate precursor miRNA (pre-miRNA) [23, 24]. The generated pre-miRNA is transported from the nucleus to the cytoplasm, where it is cleaved by the RNase III enzyme Dicer and the RNA-binding protein TRBP to yield a mature miRNA of approximately 22 nt [25, 26].

## 2. Pathogenesis of AR

AR is a nasal mucosa type I IgE mediated allergic disease. Major histocompatibility complex class II (MHCII) molecules are expressed after allergens are taken up by dendritic cells (DCs). The allergen and MHCII molecules form a complex and are presented to T cells, which differentiate into T helper 2 (Th2) cells to produce Th2 cytokines [8], such as IL-4 and IL-13. These Th2 cytokines promote the recombination of genes in the immunoglobulin constant region in B cells, which results in the secretion of allergen specific-IgE [27]; IgE binding sensitizes mast cells in the nasal mucosa. Upon repeated exposure to the same allergen, IgE is readily produced, which causes mast cells to release various inflammatory factors dominated by histamine to accelerate the synthesis of cytokines, and these cytokines act on the nasal mucosa and cause symptomatic disease [28, 29]. In addition, relevant studies have noted that cytokines that promote the differentiation of Th2 cells are also produced by type 2 innate lymphoid cells (ILC2s), which are known to produce cytokines such as IL-5 and IL-13. Therefore, ILC2s may be associated with airway allergic reactions. ILC2s affect eosinophils through the synthesis of cytokines, and this process does not require DC–antigen interactions or T-cell differentiation [30, 31]. Nasal mucus secretions from AR patients showed upregulated expression of 21 miRNAs and significantly downregulated expression of 14 miRNAs compared with those from healthy controls [32]. A total of 421 differentially expressed miRNAs were identified by an miRNA microarray analysis of nasal mucosa biopsy samples from AR patients and normal controls [33]. These data provide evidence of the role of sEVs and miRNAs in AR.

## 3. Role of sEVs and miRNAs in AR

Fang et al. [34] reported that plasma sEVs derived from patients with AR exhibit antigen-presenting characteristics and promote the differentiation of Th2 cells. Zhu et al. [35] conducted further research and found that lncGAS5 in AR epithelium-derived sEVs mediates Th1/Th2 differentiation in part by downregulating T-bet and enhancer of zeste homologue 2 (EZH2). Zhou et al. [36] showed that human mesenchymal stem cell-derived sEVs inhibit Th2 cell differentiation by regulating the miR-146a-5p/SERPINB2 pathway. Li et al. [37] determined that mesenchymal stem cell-derived sEVs containing Linc00632 inhibit Th2 cell differentiation. Fang et al. [38] found that mesenchymal stromal cell-derived sEVs could prevent ILC2-dominant allergic airway inflammation at least partly through miR-146a-5p, which inhibits Th2 cell differentiation. Luo et al. [39] found that epithelial cell-derived miR-146a in sEVs promotes the generation of IL-10-producing monocytes, which can inhibit nasal allergy.

**Figure 1:**
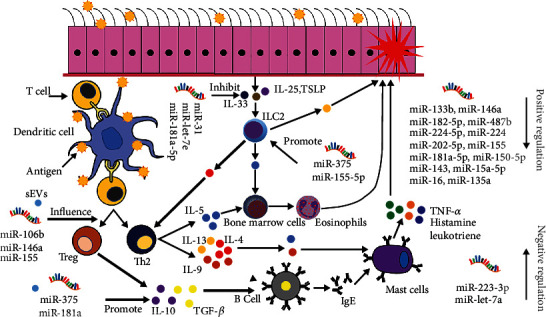
Regulation of EVs and miRNAs in allergic rhinitis.

The miRNAs are endogenous RNAs that exert posttranscriptional regulation. A previous study found differences in the expression of miRNAs in AR patients; some miRNAs are significantly upregulated or downregulated in AR patients, which indicates that miRNAs are related to the occurrence of AR. Among these miRNAs, some exert positive effects on AR [40–59], whereas others aggravate AR [60–64]. These data suggest the importance of miRNAs in the diagnosis and treatment of AR (Table 1). As shown in Figure 1, most experiments examining the regulation of AR by miRNAs show that miRNAs play a considerable role in the overall allergic inflammatory response. Most animal experiments only use the corresponding transgenic mice to stimulate AR, and miRNAs reduce AR symptoms [40–43, 46, 48, 49, 51, 52, 54, 56–60, 62]. However, the specific signalling pathways downstream of the miRNAs that affect AR remain poorly understood. These findings provide a possible direction for follow-up research and possible ideas for the precise regulation of the allergic response in the future. Most detailed studies have focused on the regulation of ILC2 and Th2 cell differentiation. Fang et al. [34] reported that miR-31 suppresses IL-13 expression and inhibits the production of thymic stromal lymphopoietin, granulocyte-macrophage colony-stimulating factor, eotaxin, and mucin 5 AC in nasal epithelial cells. Long and Zhang [55] showed that with allergen stimulation, human nasal epithelial cells prominently suppress inflammatory cytokine production through the overexpression of miR-181a-5p, which suppresses the activation of p38 MAPK signalling by negatively regulating IL-33.

## 4. Pathogenesis of NPs

Chronic rhinosinusitis (CRS) is defined as chronic inflammation of the nasal cavity and paranasal sinuses that can be classified based on the presence of nasal polyps: with nasal polyps (CRSwNP) and without nasal polyps (CRSsNP) [65]. CRS is believed to develop as a result of complex inflammatory processes that are driven by both genetic and environmental factors. It has been demonstrated that the production of mediators by various cells can monitor the immune inflammatory network underlying CRS [66]. NPs, which have a similar pathogenesis to CRS, are recognized as inflammatory lesions originating from the ethmoid sinus projecting into the nasal space. Pathogenic microorganisms are the focus of NPs research. A series of bacteria, fungi, and viruses can trigger NPs through inflammatory reactions. EBV and human papillomavirus infections are important causes of NPs, and Staphylococcus aureus and certain anaerobic bacteria are commonly identified as NPs-related pathogens [67, 68]. Immune imbalance may be an important cause of NPs. The emergence of NPs leads to destruction of the healthy environment of the nasal cavity and paranasal sinuses. Patients with NPs exhibit increased nasal mucosal epithelial permeability and decreased levels of anti-inflammatory factors which are closely related to immune dysfunction. The increased expression of type 2 cytokines is related to inflammatory factors, and inflammatory factors in eosinophils are inextricably related to the degree of oedema in polyps [6]. Some patients with NPs exhibit asthma or aspirin intolerance, which is related to the progression and severity of the NPs [69]. The incidence of NPs is high in patients who smoke; smoking negatively impacts immune function in the nasal cavities. Therefore, the nasal mucosa is in an allergic state, which results in the emergence of NPs. In addition, some studies have shown that genetic factors are involved in the development of NPs [70].

## 5. Relationship between NPs and Secreted sEVs

Recent research has shown that nasal sEVs can promote the in vitro migration of several immune cells, including monocytes, neutrophils, and natural killer (NK) cells, which further contributes to mucosal immune defence [71]. Nocera et al. found that inhaled pathogen exposure on mucosa stimulates sEVs, which send antimicrobial cargo to adjacent epithelial cells. Through this process, the sEVs secreted by the nasal mucosa and other mucosal cells strengthen the ability of the host to resist foreign infection [72].

As mentioned above, NPs are caused by tissue oedema due to an increase in the vascular endothelial gap and plasma protein overflow. The increases in angiogenesis and vascular permeability are causative factors of NPs. Previous research has indicated that sEVs and both of these biological processes can promote NPs. Some scholars have isolated sEVs from nasal lavage fluid and analysed their effects on in vitro tube formation and vascular permeability. The results showed that nasal lavage fluid-derived sEVs from patients with NPs promote tube formation by human umbilical vein endothelial cells and increase vascular permeability compared with those from unaffected people. The main secreted substance detected in this experiment is a disintegrin and metalloproteinase-10 (ADAM-10) [73], which is a protease that promotes protein release and is related to angiogenesis and permeability [74]. Other studies have found that sEVs from patients with NPs can target VE-cadherin to regulate vascular permeability through miR-22-3p [75]; moreover, relevant studies suggest that such sEVs have a significant impact on angiogenesis [76, 77]. These findings provide a new direction for studying the mechanism of NPs development in the future.

Some scholars have detected high P-glycoprotein levels in nasal mucus sEVs from patients with NPs, and the secreted sEVs effectively transport P-glycoprotein and improve the functional P-glycoprotein levels in recipient cells. An increase in P-glycoprotein expression is the key to promoting Th2-type inflammation [78]. The inhibition of P-glycoprotein can increase the accumulation of glucocorticoids in NPs tissue, which indicates that P-glycoprotein impacts the role of steroid hormones in the nasal mucosa and is a potential therapeutic marker and target of NPs [79]. In addition, some studies have reported that the coagulation system is related to the pathogenesis of NPs, but the data on the expression of coagulation-related proteins in sEVs derived from the nasal mucosa epithelium are inconsistent [80]. Another study on proteins in NPs-derived sEVs identified a series of proteins that affect cell proliferation and may lead to nasal mucosal remodelling through p35 [81], but further research is needed to clarify this finding. Researchers have concluded that quantitative analysis of the sEV proteome of patients with NPs can effectively predict the disease because changes in this proteome in NPs tissues are key to NPs growth and promote disease occurrence. This conclusion is helpful for understanding the development of NPs at the molecular level.

## 6. Role of miRNAs in NPs

**Figure 2:**
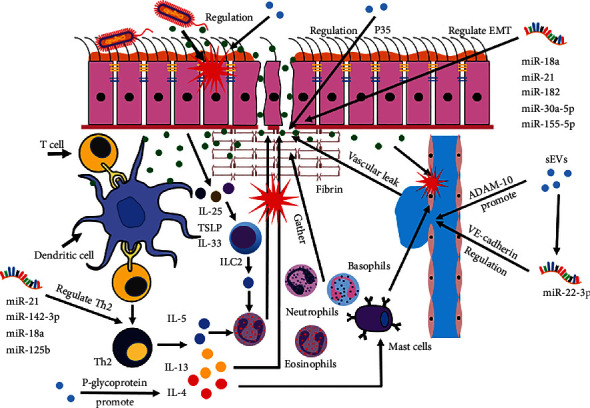
Regulation of exocrine and miRNAs in nasal polyps.

miRNAs also have a considerable impact on NPs. Relevant studies have identified differences in miRNAs in the nasal mucosa between patients with NPs and unaffected patients, which suggests a close relationship between miRNAs and the occurrence of NPs [82–84]. Among the identified miRNAs, miR-18a, miR-21, miR-125b, and miR-142-3p regulate the overall inflammatory response of the nasal mucosa [85–88], whereas miR-18a, miR-21, miR-182, miR-30a-5p, and miR-1555p regulate epithelial–mesenchymal transition (EMT) in the nasal mucosa in response to inflammatory stimulation (Figure 2) [85, 89–92]. Yang et al. [92] determined that miR-155-5p inhibitors reverse EMT of human nasal epithelial cells stimulated by TGF-*β*1, upregulate epithelial marker expression, and downregulate mesenchymal marker expression. Interestingly, Zhu et al. [64] found that miR-155-5p aggravates the secretion of Th2 cytokines by ILC2s while inhibiting the secretion of Th1 cytokines and the apoptosis of ILC2s, and these effects promote the inflammatory response of AR by targeting TP53INP1. miR-155-5p can regulate not only NPs but also AR, which indicates its potential in the treatment and diagnosis of nasal inflammatory diseases.

## 7. Summary

The occurrence and development of AR and NPs are related to sEVs and miRNAs, which regulate the pathogenesis of these diseases by controlling intercellular communication.

Many studies have revealed the effects of miRNAs and sEVs on AR, and in these studies, AR mice were used to evaluate the overall response to allergens after knockout of the corresponding miRNAs, such as the release of inflammatory factors, the serum IgE level, and the severity of allergic symptoms. However, these studies do not explain which specific processes of allergic inflammation are regulated by the miRNAs, such as antigen presentation by dendritic cells, the differentiation of naïve T cells, or the degranulation of mast cells, which are also the key points that should be analyses in future research.

Previous studies have confirmed the differences in miRNAs expression in the nasal mucosa and mucus of patients with AR and genetic materials of sEVs, and the findings provide ideas for further experiments. However, based on the existing research on sEVs and miRNAs, they can be transmitted between cells, that is, through the blood, nasal mucosa, and nasal secretions. sEVs and miRNAs at the three positions can be transmitted to each other to regulate AR, but no studies have investigated these three positions simultaneously. Subsequently, nasal mucosa, nasal mucus, and even blood samples from patients with AR and NPs and the control group should be collected at the same time, and relevant miRNAs sequencing can be performed. The regulatory roles of miRNAs in AR and NPs may be further clarified by analysing the differences between the three groups. If some specific substances in an miRNAs or sEVs are specific or highly expressed in patients with AR or NPs, sEVs in nasal mucus can be effectively used in the diagnosis of AR and NPs as a noninvasive and quantitative index.

If some studies clarify that the miRNAs or some sEV substances can treat AR or NPs, sEVs and miRNAs are expected to play a role in future targeted drug therapy due to their nanocarrier properties. AR and NPs can be effectively treated for a long time through sustained-release nanomaterials coated with the corresponding miRNA and sEVs. These ideas warrant clinical attention.

## Figures and Tables

**Table 1:** miRNAs that regulate allergic rhinitis. HNECs: human nasal epithelial cells, BMDCs: bone marrow-derived dendritic cells, PBMCs: peripheral blood mononuclear cells.

miRNAs and related proteins	Activity	Experimental model
miRNAs that positively regulate AR		
miR-133b, Nlrp3 [40]	miR-133b reduces the concentration of ova-specific IgE, AR symptoms, and cytokine levels in AR mice by inhibiting NLRP3 inflammasome-mediated inflammation.	Mice
miR-146a, TLR4, TRAF6, NF-*κ*B [41]	The miR-146a mimic significantly reduces AR symptoms and allergen-specific IgE and inflammatory cytokine levels in AR mice and reduces the levels of cytokines released by Th2 cells.	Mice
miR-182-5p, TLR4/NF-*κ*B [42]	The miR-182-5p mimic reduces symptoms in AR mice and the number of inflammatory cells in alveolar lavage fluid and the levels of inflammatory factors in serum but increases IFN-*γ* and IL-2 release.	Mice
miR-487b, IL-33/ST2 [43]	The upregulation of miR-487b in AR mice leads to reductions in IgE and proinflammatory cytokines as well as pathological changes.	Mice
miR-31, IL-13 [44]	miR-31 improves AR by inhibiting IL-13-induced nasal epithelial inflammatory responses.	Mice, HNECs
Let-7e, JAK1/STAT3 [45]	The overexpression of the miRNA let-7e decreases cytokine levels in AR mice and IL-13-stimulated nasal epithelial cells.	Mice, HNECs
miR-224-5p, TLR4, MyD88, NF-*κ*B [46]	The overexpression of miR-224-5p reduces the levels of inflammatory cells in nasal lavage fluid and the levels of proinflammatory factors in serum and nasal mucosa of AR mice.	Mice
miR-106b, Egr-2 [47]	miR-106b negatively regulates the allergenicity and Th2 polarization of bone marrow-derived dendritic cells after allergen stimulation.	BMDCs, CD4^+^ T cells
miR-224, CDK9 [48]	The upregulation of miR-224 reduces AR symptoms and the levels of cytokines, IgE, and histamine. In addition, miR-224 reduces inflammatory cell infiltration and excessive secretion by glands in the nasal mucosa.	Mice
miR-202-5p, MATN2 [49]	High expression of miR-202-5p is elevated in PBMCs, CD4+ T cells, and Tregs of AR patients, and miR-202-5p promotes Treg differentiation via MATN2.	PBMCs
miR-375, JAK2, STAT3 [50]	Injection of the miR-375 inhibitor after allergen sensitization reduces the IL-6, IL-10, and TNF-*α* levels, and the overexpression of miR-375 reverses the TNF-*α*-mediated apoptosis of nasal mucosal cells.	Mice
miR-155, c-Maf [51]	miR-155 regulates the production of Th2 cytokines, airway inflammation, and allergic symptoms in AR mice.	Mice
miR-181a-5p, HMGB1 [52]	miR-181a-5p overexpression reduces the binding between HMGB1 and RAGE by inhibiting HMGB1, which alleviates the Treg/Th17 immune imbalance and prevents AR from developing into asthma.	Mice
miR-146a [53]	Further analysis shows that miR-146a induces transforming growth factor-*β* expression in dendritic cells and then promotes the differentiation of naïve CD4^+^ T cells into Treg cells.	Mice
miR-150-5p, ICAM-1, p38 [54]	Treatment with miR-150-5p lentivirus reduces the symptoms, nasal mucosal inflammation, and levels of serum type 2 cytokines, ova-specific IgE, and ILC2s in AR mice.	Mice
miR-181a-5p, IL-33/p38 MAPK [55]	miR-181a-5p negatively regulates IL-33, inhibits the activation of p38 MAPK signalling, and reduces allergic inflammation.	RPMI2650 cells
miR-143, IL13R*α*1 [56]	miR-143 significantly decreases the mRNA and protein levels of GM-CSF, eosinophil chemokines, and MUC5AC in IL-13-stimulated NECs.	HNECs
miR-15a-5p, JAK2 [57]	IL-13 significantly upregulates the secretion of eosinophil chemokine-1, GM-CSF, and MUC5AC by endothelial cells in vitro, upregulates the expression of ANRIL and JAK2, and downregulates the expression of miR-15a-5p.	HNECs
miR-16, IKK*β*, NF-*κ*B [58]	In IL-13-treated nasal epithelial cells, the upregulation of miR-16 decreases the GM-CSF, eosinophil chemokine, and IL-1*β* levels and the mRNA and protein levels of IL-6, IL-10, and MUC5AC.	HNECs
miR-135a, GATA-3 [59]	The intranasal administration of miR-135a in AR mice significantly decreases, the total serum IgE concentration, and miR-135a inhibits the infiltration of eosinophils and mast cells into the nasal mucosa in AR mice; these processes are regulated by GATA-3.	Mice
miRNAs that negatively regulate AR		
miRNA-223-3p, INPP4A [60]	The upregulation of miR-223-3p in AR mice significantly increases the allergen-specific IgE concentration, AR symptoms, proinflammatory cytokine levels, and eosinophil infiltration in the nasal mucosa.	Mice
miR-375, TSLP [61]	TSLP expression is significantly increased in HNECs transfected with the miR-375 mimic, which also promotes ILC2s to produce type II cytokines, and the miR-375 inhibitor alleviated allergic symptoms and type II cytokine production in AR mice.	Mice, HNECs
miR-let-7a, OPN [62]	Mice treated with the miRNA let-7a present significantly worse AR symptoms, a greater number of eosinophils, and increased goblet cell hyperplasia in the nasal mucosa.	Mice
miR-181a, miR-155 [63]	miR-181a upregulates IL-10 and TGF-*β* expression, and miR-155 promotes the differentiation of Tregs from CD4^+^ T cells.	Mice, PBMCs
miR-155-5p, TP53INP1 [64]	miR-155-5p promotes the secretion of Th2 cytokines by ILC2s and inhibits the secretion of Th1 cytokines and the apoptosis of ILC2s.	ILC2s

## Data Availability

There are no relevant data.
